# Notes and News

**Published:** 1902-08-16

**Authors:** 


### Notes and News.

With the view of securing universal vaccination in the British Empire, an Imperial Vaccination League has been formed under the presidency of the Duke of Fife and with a powerful committee. The office of the league is at 53 Berners Street.

Lord Rosebery endeavoured unsuccessfully to introduce an amendment into the Licensing Bill of Inebriate Homes, before the third reading. Holding the opinion that female inebriety is practically incurable, he endeavoured to prevent an applicant husband being denied a separation order from his inebriate wife, in favour of an attempt being made to cure her in a home.

An attempt is being made in Russia to form a standard of payment for medical attendants. Etiquette has not formed any standard of charges in Russia, and the discrepancies are most marked between the remuneration obtained by the celebrated members of the profession and their more obscure brethren—a condition of affairs not entirely unknown in this country. We venture to doubt the utility, and indeed the practicability, of all such standards.

The result of the prize competition for the planning of the King's Sanatorium for Consumption has been made known. Dr. Arthur Latham, Assistant Physician to the Brompton Hospital for Consumption and St. George's Hospital, has been awarded the first prize. The second prize falls to Dr. F. Wethered, also an Assistant Physician at the Brompton Hospital. The third has been awarded to Dr. E. Morland, House Physician to the Hospital for Sick Children, Great Ormond Street. Honourable mention is awarded to the following essay writers for the great excellence of their work. Dr. P. S. Hichens, formerly Assistant Resident Medical Officer at the Brompton Hospital for Consumption; Dr. Turban, of Davos; Dr. Jane Walker, Superintendent of the East Anglian Sanatorium for Consumption; and Dr. J. Wills, of Bexhill. All the successful competitors were associated with architects. The advisory committee were Sir William Broadbent, Sir Douglas Powell, Sir Felix Lemon, Dr. Hermann Weber, and Dr. Theodore Williams.

The Prussian State Railways are introducing 78 railway ambulance carriages upon their system for use in cases of accident upon the line. They will be kept always ready for use, and be supplied with all things necessary for operations and dressings.

The report of Mr. Sharpe, Commissioner of the British Central Africa Protectorate, shows that under the various administrations covered by his report malaria is decreasing amongst Europeans. This is mainly attributed to the greater care which is now taken to avoid mosquito bites.

The prospects for the season in Egypt are rather better than they were, in that the outbreak of cholera has not taken on that explosive and widespread character which was anticipated by some. But there are still a considerable number of cases, and while that is so it is far too early to talk of safety.

No definite conclusions as to the result of inoculation for enteric fever have yet been arrived at, and a report on the effect of inoculation during the South African war is not yet issued. In India the figures are reported to be in favour of the experiment, but no statistics have, as yet, been collected showing the period during which such immunity as may be produced by the process remains effective.

Dr. Garnault is satisfied, up to the present time, with the results of his experiment at self-inoculation with the virus of bovine tuberculosis. He rejoices in the presence of cells characteristic of tuberculosis on his arm, but regretfully admits that the true bacillus of tuberculosis has not yet shown its presence. The enemy, otherwise Dr. Koch's supporters, on the other hand, are unmoved by the experiment, as they say that the test should have been confined to drinking milk, as Dr. Koch's contention was that the contagion was not conveyed by ingestion. They contend that the experiment, even if wholly successful in showing definite contagion by inoculation, will prove nothing unless Dr. Garnault ascertained that he himself was free from tuberculosis before the experiment.

The West of England is awakening to the necessity of active effort to cope with the ravages of consumption among the people. Recently the Devon and Cornwall Association for the Prevention of Tuberculosis decided to acquire the Didworthy house and estate of 67 acres, situated about two miles from South Brent, and meeting every requirement for an open-air sanatorium for the treatment of poor, non-paying patients. At the outset it is contemplated providing 15 beds, and it is calculated that 45 patients could be treated annually. Towards the cost over £4,000 has been obtained in subscriptions (including an anonymous donation of £3,000) by the Committee of which Dr. Clay and Mr. Carlile Davis, of Plymouth, are the chairman and hon. secretary respectively, with Mr. Thomas Bulteel hon. treasurer. The annual cost of maintenance will be anything from £1,000 to £1,500 per annum, and the success of the institution must depend greatly on the generosity of the public or the contributions of public bodies.

